# Bayesian hierarchical stock–recruitment models for setting conservation limits for Atlantic salmon stocks in Scotland

**DOI:** 10.1111/jfb.70363

**Published:** 2026-02-18

**Authors:** James P. Ounsley, Nora N. Hanson, Gordon W. Smith, Jonathan P. Gillson, Brian A. Shields, Stuart J. Middlemas

**Affiliations:** ^1^ Scottish Government Marine Directorate Freshwater Fisheries Laboratory Pitlochry UK; ^2^ Scottish Government Marine Directorate Freshwater Fisheries Laboratory Field Station Montrose UK; ^3^ The Centre for Environment, Fisheries and Aquaculture Science Lowestoft UK; ^4^ Environment Agency Richard Fairclough House, Knutsford Road Warrington UK

**Keywords:** biological reference points, cross‐validation, fisheries management, *Salmo salar*, stock and recruitment

## Abstract

Atlantic salmon (*Salmo salar* L.) populations in Scotland are subject to active management and conservation practices which require biological reference points (BRPs), specifically conservation limits, defined at the level of the stock. Acquiring the data necessary to independently derive these BRPs for all managed populations in Scotland is prohibitive, motivating the use of Bayesian hierarchical stock–recruitment models. These models provide a framework for the joint analysis of multiple monitored stocks, and the transportation of BRPs to non‐monitored stocks. This framework was adapted to introduce nationally relevant and available covariates that might explain variation in recruitment dynamics among stocks and reduce uncertainty in posterior predictions of BRPs. Model selection was designed to maximise the prediction of BRPs for new stocks via leave‐one‐group‐out cross‐validation. Out‐of‐sample predictive performance was maximised by including information on latitude, land usage within the catchment and historic catch per area of salmon habitat in the model. The extensions to Bayesian hierarchical stock–recruitment methods presented here, when applied at a national scale, result in more locally discriminative posterior predictions compared to existing methods and are readily applicable to other stocks and species.

## 1 INTRODUCTION

Atlantic salmon (*Salmo salar* L., hereafter salmon) stocks are important cultural and economic resources throughout their range. The observed declines in stock size across the North Atlantic (ICES, 2021; Olmos et al., 2020) have focused attention on management and conservation practices both at national and international levels. Biological reference points (BRPs) are widely used to assess the status of stocks and determine whether management action is required to meet the overarching goal of preserving salmon resources for future generations (Potter et al., 2003). The North Atlantic Salmon Conservation Organisation (NASCO) has produced international best practice guidelines that recommend using the number of spawners required to achieve long‐term maximum sustainable yield (MSY) as a BRP for salmon stocks (NASCO, 1998).

The number of spawning adult salmon required to achieve MSY, $S^{*}$, is typically derived by fitting stock–recruitment (SR) models to time‐series of salmon abundance (e.g., Hindar et al., 2011; Ó Maoiléidigh et al., 2004; Prévost et al., 2003; White et al., 2016). Time‐series of SR data are available where long‐term monitoring programmes provide estimates of the spawning stock and subsequent recruitment, although the collection of sufficient data to allow fitting of SR models is time consuming and expensive. Such efforts are therefore limited to a subset of the total number of stocks, and a major challenge is transferring BRPs from these stocks to those without SR data.

Previous studies investigating European stocks at a large spatial scale have sought to transfer BRPs to data‐poor areas using a Bayesian hierarchical stock–recruitment (BHSR) modelling framework (Prévost et al., 2003; White et al., 2016). The BHSR framework, outlined by Prévost et al. (2003), builds on prior meta‐analytical approaches (Liermann & Hilborn, 1997; Myers, 2001) that pool information and uncertainty across SR datasets, providing a balance between the assumption of complete independence between stocks and modelling all stocks in aggregation (e.g., Potter et al., 1998). Crucially, the BHSR approach provides a natural method to model the variation in $S^{*}$ among stocks conditional on explanatory covariates, allowing the prediction of $S^{*}$ for stocks without SR data. Previous analyses of European Atlantic salmon stocks have used latitude, a proxy for productivity, as a simple linear predictor of $S^{*}$. However, despite identifying a broadly positive relationship, a considerable amount of among‐stock variation in $S^{*}$ remains unexplained (Prévost et al., 2003; White et al., 2016).

The productivity of salmon stocks is influenced by many biotic and abiotic factors operating at a range of spatial scales (Ugedal & Finstad, 2011). Ideally such factors would be compared with the previously identified relationship with latitude (Prévost et al., 2003; White et al., 2016) to identify potential improvements to out‐of‐sample prediction. This requires accommodation of additional explanatory variables within the BHSR framework. Although it is desirable that models take advantage of all relevant explanatory variables, any such approach runs the risk of over‐fitting on stocks with available SR data, undermining the ability for any resultant model fit to generalise to out‐of‐sample stocks appropriately. This motivates the use of model comparison and selection techniques that consider the hierarchical and spatial structure of the data (Roberts et al., 2017) and are tailored to best meet the desired outcomes (Wenger & Olden, 2012). For example, when dealing with grouped data, a naive k‐fold cross‐validation (CV) approach in which data points are randomly assigned to each fold does not guarantee independence between the training data and the held‐out data with respect to those groups. Violating this independence is likely to result in an overestimate of the predictive ability of a model when applied to new groups, and model selection may favour too complex models (Roberts et al., 2017).

The present study is concerned with the SR dynamics of salmon stocks across Scotland where spatial variation in climatic, geomorphological and biogeographic patterns manifests in considerable diversity of hydroclimatological regimes and riparian habitat for its rivers (Soulsby et al., 2009). This diversity has been shown to impact juvenile salmon densities in streams (Malcolm et al., 2019) and is likely to play a role in SR dynamics. Such patterns are not likely to be adequately captured by the latitudinal relationship previously identified in European stocks. Therefore, the present study used the BHSR framework to determine the most suitable explanatory variables for the transportation of BRPs in Scotland, with the specific aim of avoiding overfitting by maximising out‐of‐sample predictive performance.

## 2 METHODS

### 2.1 Data

Stock–recruitment relationships for salmon are usually expressed in egg numbers per square metre of river habitat (e.g., Prévost et al., 2003; White et al., 2016) to account for variation in catchment size among stocks. Such relationships can be fitted to various points of the salmon life cycle, depending on the availability of data on, for example, the number of emigrating smolts and returning adult salmon (Hindar et al., 2011). To maximise the number of datasets available the present analysis used information from rivers and tributaries for which appropriate abundance data were available to produce egg‐to‐egg SR series. Note that estimates of $S^{*}$ are dependent upon the part of the life cycle at which recruitment is considered; $S^{*}$ derived from egg‐to‐egg SR series will differ from those based on recruitment at pre‐fishery abundance, for example. The numbers of spawning salmon were derived by adjusting counts of adult salmon moving upstream through fish counters or traps (e.g., Glover & Malcolm, 2018; Thorley et al., 2005) and removing mortalities associated with fisheries (e.g., Webb, 1998) and natural causes (Milner et al., 2000). The number of female spawners was estimated by assuming a proportion of 0.5 one‐sea‐winter (1SW) and 0.7 multi‐sea‐winter (MSW) fish were female (Scottish Government Marine Directorate unpublished data). Egg deposition estimates were derived using previously established relationships (Hanson et al., 2020) and the best available information on the size and age of females at each site. Age information was also used to assign the brood year for each age class of spawners allowing the number of recruits associated with spawning years to be estimated. Reconstructed time‐series, expressed as total eggs, were available for 11 catchments in Scotland (referenced here by fish counter/trap location) and 1 from the northwest of England (the Lune). Total egg numbers were then standardised by accessible wetted area.

Wetted areas were derived from the Ordnance Survey (OS) MasterMap (https://docs.os.uk/os-downloads/products/maps-and-imagery-portfolio/os-mastermap-topography-layer) digital mapping dataset. Water features where salmon were known to be present were extracted from this dataset based on the distribution of salmon [Gardiner and Egglishaw (1986) and subsequent updates]. Areas associated with the polygons upstream of the counters/traps were summed to determine the total wetted area of salmon habitat (Table 1).

**TABLE 1:** Summary of available stock–recruitment data and among‐stock explanatory variables arranged by latitude.

Stock	*N*	Year range	Wetted area ($m^{2}$) (WA)	Lat.	Lon.	Spatial position (SP)	Catch per area (CPA)	Catchment type (CT)	Land usage (LU)
Helmsdale	10	2001:2010	1,194,674	58.1	−3.6	3.6	14.1	Whole	−0.42
Beauly	19	1991:2009	1,693,203	57.5	−4.4	3.0	4.7	Whole	0.05
Moriston	33	1969:2009	1,046,781	57.2	−4.7	2.7	4.5	Upper	0.10
Morar	30	1963:1998	227,824	57.0	−5.8	5.8	1.9	Whole	−0.01
Girnock	43	1966:2009	62,781	57.0	−3.1	2.5	8.1	Upper	−0.39
Baddoch	22	1988:2009	40,253	56.9	−3.4	2.4	8.1	Upper	−0.20
North Esk	30	1981:2010	2,607,790	56.8	−2.4	2.3	6.8	Whole	0.06
Westwater	20	1991:2010	535,076	56.8	−2.6	2.3	6.8	Whole	−0.13
Pitlochry	25	1986:2010	2,302,241	56.7	−3.7	2.2	5.2	Upper	−0.06
Awe	46	1965:2010	2,167,343	56.4	−5.2	6.3	3.3	Whole	0.44
K. Dee	4	2007:2010	1,643,215	54.8	−4.0	7.9	0.4	Whole	0.55
Lune	9	1998:2006	4,227,300	54.1	−2.7	8.7	2.7	Whole	0.47

*Note*: Details about the data and explanatory variables are provided in the main text and Supplementary Material in the case of spatial position.

The River Lune stock was included in the analysis because this watercourse is situated on the west coast of England, close to the Kirkcudbrightshire (K.) Dee, a stock with only four available SR datapoints. It was assumed that the inclusion of the Lune data would provide additional information on the otherwise underrepresented area of the parameter space in which the K. Dee exists (Table 1). Importantly, although the Lune is included in this analysis, this model is focused on deriving CLs for rivers in Scotland only. For management purposes, any analysis of the Lune should include additional information from England and Wales.

**FIGURE 1:**
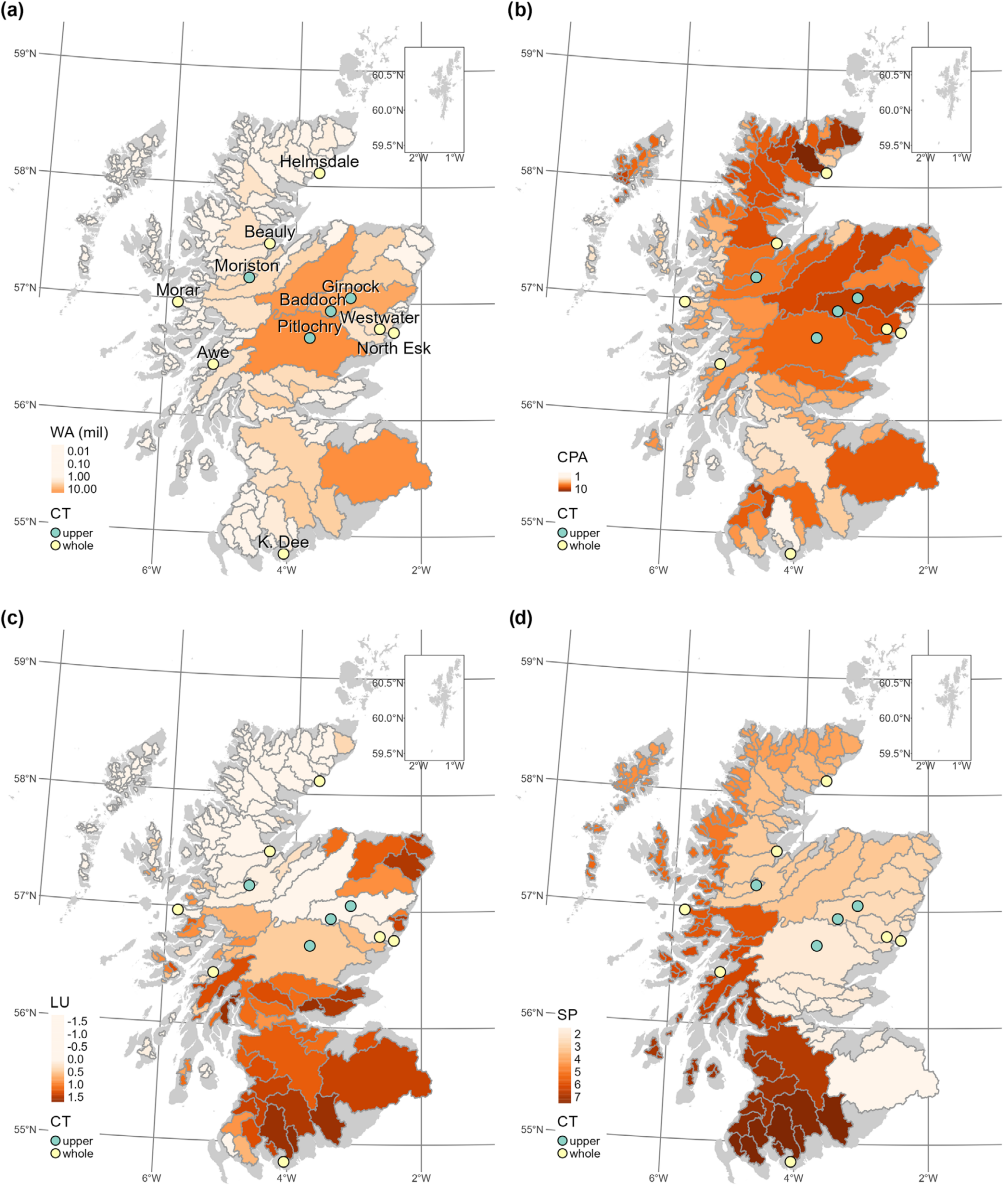
Mapped covariates including wetted area (WA) (a), (log) catch per area (CPA) (b), land usage (LU) (c) and spatial position (SP) (d) for all assessment areas. Points indicate counter or trap location of Scottish monitored stocks coloured as a whole or upper catchment type (CT). Salmon Stock Assessment Area boundaries are derived from the Scottish Environment Protection Agency (SEPA) 1:50,000 river catchment data. Based on digital spatial data licensed from the UK Centre for Ecology and Hydrology, © NERC (CEH). Contains Ordnance Survey data © Crown copyright and database right 2024.

The following set of explanatory variables were considered as candidates to capture among‐stock variation in BRPs. In order for these covariates to be of use for out‐of‐sample prediction, they were limited to those data that were available for all stocks of concern for fisheries management in Scotland, not just those with SR data (Figure 1).

- Catch per area (CPA): Historical information on rod catches (retained and released) of salmon was used as a proxy for the relative productive capacity of the catchments considered in the present analysis. Scotland‐wide catches from rod fisheries in 109 fishery districts were available for the period 1952–2016 (Marine Scotland, 2017a, 2017b). CPA was defined as the 80th percentile of the time‐series divided by the area of salmon habitat. Where multiple stocks fall within the same fishery district, each stock is assigned the same value. The log of the CPA was taken to improve linearity in the model.
- Land usage (LU): Information on land cover was retrieved from the 2015 Land Cover Map produced by the UK Centre for Ecology and Hydrology (Rowland et al., 2017). Land cover was divided into the proportion of 10 different land use types in each 1‐km grid square covering the UK. This information was used to estimate the proportion of each land‐use type in each of the catchments. A principal components analysis (PCA) was used to reduce the 10 different variables into the first principal component, which explained 60% of the variation in land use. Retained loadings of the first principal component broadly contrast assessment areas between mountainous and grassland regions (Supplementary Material).
- Latitude (Lat): The latitude of the fish trap/counter (or river mouth for out‐of‐sample prediction) is a readily accessible and informative proxy for hydroclimatological variation related to BRPs for salmon (Prévost et al., 2003; White et al., 2016).

Each of these continuous linear explanatory variables (CPA, LU, Lat) was scaled and centred to aid in model fitting and interpretation of relative effect sizes.

Data on the Baddoch, Girnock, Pitlochry (Tummel) and Moriston stocks were for upper river catchments. These upper catchments tend to consist of salmon returning to rivers early in the year (Laughton & Smith, 1992). The existence of subpopulations at such scales is supported by tagging studies and genetic analysis undertaken at the smallest of these tributaries, the Baddoch and Girnock, both of the River Dee (Verspoor, 1997). However, straying rates for tributaries may be higher than that of whole catchments, which could result in greater year‐to‐year variance in recruitment for these stocks. A fixed effect of catchment type (CT) was included in all models to control for differences in recruitment between upper catchment and full catchment stocks.

Four of the stocks in the dataset have a strong dependency because they inhabit the same river as another stock included in the analysis. The Girnock and Baddoch are stocks from separate tributaries on the Aberdeenshire Dee. The Westwater is a sub‐stock on a major tributary of the North Esk, located approximately 7 miles from the river mouth accounting for approximately 25% of the spawning stock and 20% of the total habitable area. Given that in each case only two stocks have the shared river dependency, this cannot be accounted for through additional hierarchies in the modelling process. However, these dependencies are partially captured through similar and shared explanatory variables.

An alternative approach to capturing among‐stock variation in BRPs is to assume a non‐linear spatial structure in the data (Su et al., 2004). In addition to the covariates detailed above, a spatial position (SP) metric that captured the coastal proximity of the river mouths supporting each stock was constructed (Figure 1). This covariate was used as the basis of a penalised regression spline included in the BHSR model as an optional additive component. Full details of the SP covariate derivation and spline implementation are provided in Supplementary Material.

### 2.2 Bayesian hierarchical stock–recruitment model

The BHSR methodology of Prévost et al. (2003) was adapted to include additional covariates. To allow direct estimation of the BRPs, the Schnute and Kronlund (2002) form of the Ricker function was used, relating the recruitment $R_{i,g}$ in year i for stock g to the associated spawning stock $S_{i,g}$ as follows:

$$\text{Ricker}\left(S_{i,g},S_{g}^{*},h_{g}^{*}\right)=\frac{S_{i,g}}{1-h_{g}^{*}}\exp\left[h_{g}^{*}\left(1-\frac{S_{i,g}}{S_{g}^{*}}\right)\right].$$

where $S_{g}^{*}$ and $h_{g}^{*}$ are, respectively, the spawning stock and harvest rate at MSY for stock g. The recruitment at MSY, $R^{*}$ is then derivable as

$$R^{*}=\frac{S^{*}}{\left(1-h^{*}\right)}.$$

The model was assumed to have independent log‐normal process error with the standard deviation σ constant across all stocks giving,

$$R_{i,g}\sim \text{LogNormal}\left(\log\left(\text{Ricker}\left(S_{i,g},S_{g}^{*},h_{g}^{*}\right)\right),\sigma \right).$$

### 2.3 Incorporating explanatory variables

$S^{*}$ and $h^{*}$ were modelled within a hierarchical structure, with the value for each stock being drawn from a common distribution conditional on among‐stock explanatory variables. Among‐stock variation in $S^{*}$ was assumed to be log‐normally distributed with,

(1) $$\log\left(S_{g}^{*}\right)∼\text{Normal}\left(\beta^{S}X_{g},\sigma^{S}\right),$$

where $X_{g}$ and $\beta^{S}$ are the design matrix and corresponding coefficients for an intercept and linear among‐stock explanatory variables (CPA, LU, CT and Lat).

Among‐stock variation in $h^{*}$ was modelled in a similar way, using a logit link transformation to bound $h^{*}$ between 0 and 1,

(2) $$\begin{array}{c} \text{logit}\left(h_{g}^{*}\right)∼\text{Normal}\left(\beta^{h}X_{g},\sigma^{h}\right), \end{array}$$

where $\beta^{h}$ is the coefficient for the explanatory variables on $h^{*}$. Note that $X_{g}$ is shared in the definition of $S^{*}$ and $h^{*}$. This logistic formulation for $h^{*}$ was found to be more stable during model fitting than the beta distribution as used by Prévost et al. (2003).

To fully define the model, priors were specified for σ and the set of hyperparameters ($\beta^{S},\beta^{h},\sigma^{S}$, and $\sigma^{h}$, Table 2). A weakly informative standard normal prior was assigned to each coefficient acting on the among‐stock explanatory variables. For the variance parameters, a half‐Cauchy prior was used (Gelman, 2004).

**TABLE 2:** Priors on parameters and hyper parameters in the model.

(Hyper) parameter	Bounds	Prior
$\beta^{S}$	+/− inf.	Normal (0,1)
$\beta^{h}$	+/− inf.	Normal (0,1)
$\sigma^{S}$	0, +inf.	Half‐Cauchy (0,1)
$\sigma^{h}$	0, +inf.	Half‐Cauchy (0,1)
σ	0, +inf.	Half‐Cauchy (0,1)

### 2.4 Candidate models

A set of candidate models was constructed by including different explanatory variables in $X_{g}$. All possible linear combinations of the among‐stock explanatory variables, CPU and LU, (excluding interactions and including an intercept plus CT only model) were paired with either no spatial explanatory variable, the inclusion of a linear term for Lat in $X_{g}$ or the penalised regression spline based on SP (Supplementary Material). This resulted in a set of 4×3=12 candidate models.

### 2.5 Selecting the best predictive model

The out‐of‐sample predictive performance of the models was assessed using a blocked CV strategy (Roberts et al., 2017; Wenger & Olden, 2012). Given the limited number of stocks in the dataset, blocks were constructed on a stock‐by‐stock basis resulting in a 12‐fold CV, with each fold composed of data from a single stock (i.e., ‘leave‐one‐stock‐out’ CV). This approach ensured that model performance was evaluated based on the accuracy of predictions for stocks not included in the data used to fit the model, which is ultimately what is desired when transporting BRPs to new stocks.

The CV score for a model was quantified using the log predictive density (Gelman et al., 2014; Vehtari et al., 2017). Let θ be the set of parameters in the model, $y_{g}$ be the subset of the data belonging to stock g=1,…,G and $y_{-g}$ be the training set excluding data for stock g, then the joint log predictive density for all data points $y_{g}$, belonging to stock g is

$$\log\;p\left(y_{g}|y_{\left(-g\right)}\right)=\log\;\prod_{i\in g}\int p\left(y_{i}|\theta \right)p\left(\theta |y_{-g}\right)\mathrm{d\theta},$$

Given MCMC samples from the posterior parameter distribution θ, labelled $\theta^{s}$ with s=1,…,S, the expected log predictive density for a new stock g is given by

$${\hat{\text{elpd}}}_{g}=\log\left(\frac{1}{S}\sum_{s=1}^{S}p\left(y_{g}|\theta^{s}\right)\right).$$

Taking the mean over all stocks, we derive the cross‐validation score

$${\hat{S}}_{\mathrm{CV}}=\frac{1}{G}\sum_{g=1}^{G}{\hat{\text{elpd}}}_{g}.$$

This method uses a marginal focus, effectively treating each group as a single data point (Gelman et al., 2014; Vehtari et al., 2017; Yates et al., 2023). Models with higher values of ${\hat{S}}_{\mathrm{CV}}$ have greater out‐of‐sample predictive accuracy.

CV was performed by fitting all models using Hamiltonian MCMC methods in Stan (http://mc-stan.org/) with subsequent analysis conducted in R 3.3.4 (http://www.r-project.org/). For each fold, 15,000 post‐warmup samples across three chains were generated after a warmup of 5000 iterations per chain. For all fits, diagnostic checks were performed; for all unknown parameters convergence was assessed with the Gelman‐Rubin statistic, ensuring an $\hat{R}$ within 0.05 of 1 (Gelman & Rubin, 1992), and a minimum effective sample size of 1000 per parameter was ensured (Gelman et al., 2003).

Model selection was performed using a modified one‐standard‐error rule (Yates et al., 2021). This approach aims to avoid overfitting by selecting the best performing but least complex model taking into account uncertainty in ${\hat{S}}_{\mathrm{CV}}$ and between model covariance in ${\hat{\text{elpd}}}_{g}$. Following Yates et al. (2021), given the model with the highest ${\hat{S}}_{\mathrm{CV}},$ $m^{\max}$, and a candidate model $m^{k}$ the modified standard error for $m^{k}$, $\sigma_{\mathrm{mod},k}$, is defined:

$$\sigma_{\mathrm{mod},k}=\sigma_{\max}\sqrt{1-\rho_{\max,k}},$$

where $\sigma_{\max}$ is the standard error in ${\hat{S}}_{\mathrm{CV}}$ for $m^{\max}$ and $\rho_{\max,k}$ is the covariance in ${\hat{\text{elpd}}}_{g}$ between models $m^{\max}$ and $m^{k}$. The model selection rule is then to select the least complex model within one modified standard error of the ${\hat{S}}_{\mathrm{CV}}$ of $m^{\max}$, denoted $m^{*}$. If two models are similar in ${\hat{S}}_{\mathrm{CV}}$ but are highly correlated in pointwise likelihood, only the strictly best model is likely to be considered.

After identifying $m^{*}$, this model was refit on the entire dataset with three MCMC chains and 75,000 post‐warmup samples over all chains. As with the CV, this final model was assessed for convergence and effective sample size. Details of the residuals and posterior summaries of all unknowns are presented in Supplementary Material in Data S1. Some evidence of non‐constant variance between stocks was apparent and is also addressed in Supplementary Material in Data S1.

This fit was used to derive $S^{*}$ and $h^{*}$ for 167 out‐of‐sample Scottish stocks using the appropriate values of explanatory variables. Where these out‐of‐sample stocks included monitored upper‐catchment sub‐stocks, the fitted values were not used and new values were predicted at the whole catchment level.

## 3 RESULTS

### 3.1 Model selection

**FIGURE 2:**
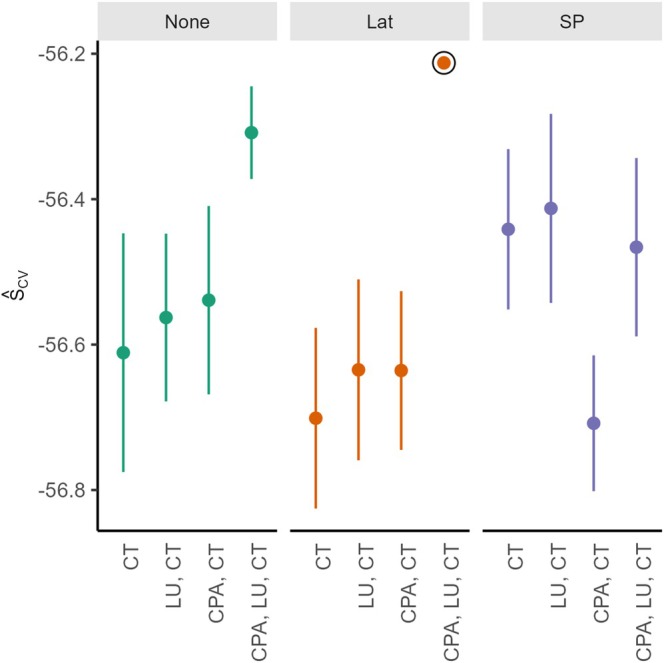
${\hat{S}}_{\mathrm{CV}}$ for candidate models +/− one correlation adjusted standard error in the difference to $m^{max}$ . Circle denotes models within one adjusted standard error of $m^{max}$ . CT, catchment type; CPA, (log) catch per area; LU, land usage; SP, spatial position. All models include an intercept term.

There were no models within one correlation adjusted standard error of the ${\hat{\;S}}_{\mathrm{CV}}$ of $m^{\max}$, as such $m^{*}=m^{\max}$ (Figure 2). The selected model included both continuous covariates LU and CPA, along with latitude as a spatial covariate. The next best model also included LU and CPA, but no spatial component. For the latitudinal and none spatial models, model performance generally increased with model complexity, and this was not the case for the models with the non‐linear spatial component where the inclusion of CPA reduced model performance.

**FIGURE 3:**
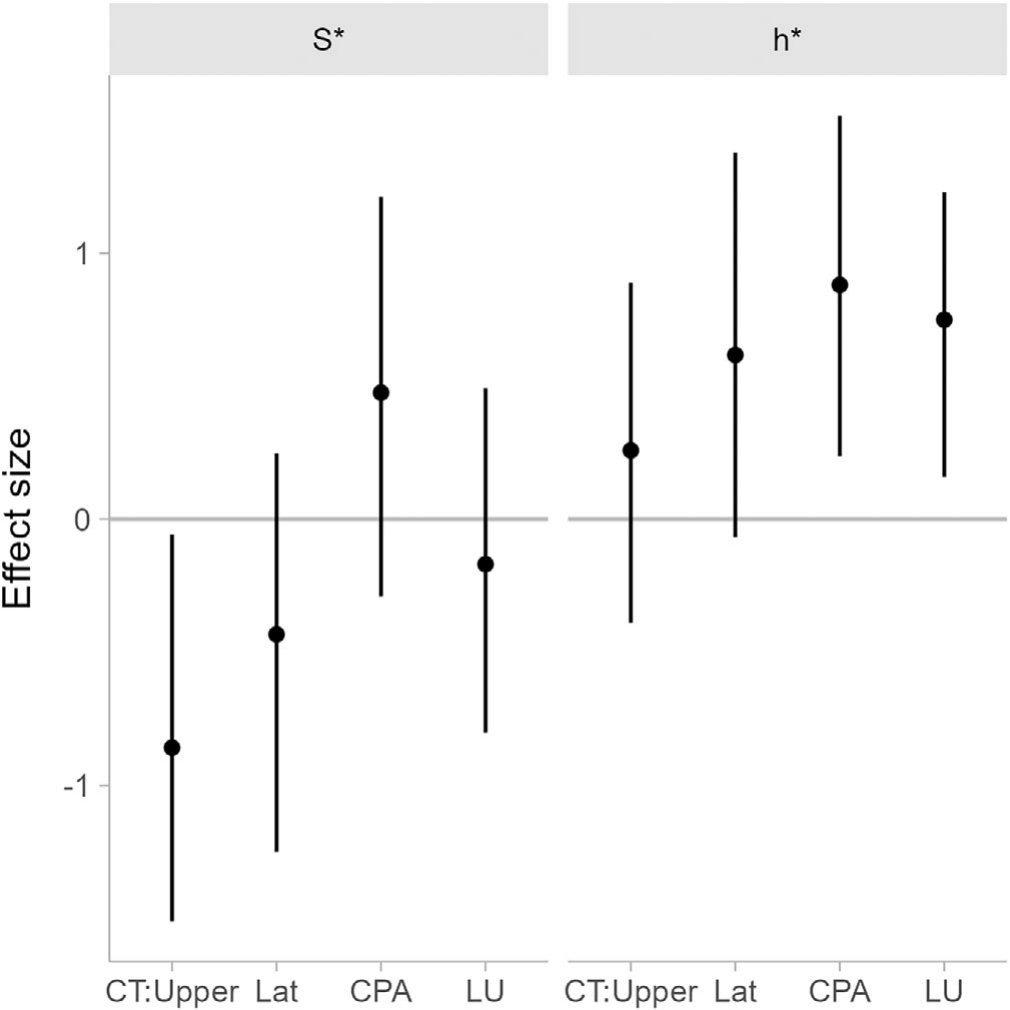
Median and 90% credible intervals of coefficients on the linear predictors of spawning stock ($S^{*}$) and harvest rate at maximum sustainable yield (MSY) ($h^{*}$) for $m^{*}$. CT:Upper, catchment type effect for upper catchment stocks; Lat, latitude; CPA, (log) catch per area; and LU, land usage.

Figure 3 shows the effect sizes of the covariates retained in $m^{*}$ for both $S^{*}$ and $h^{*}$. The largest effect size for covariates on $S^{*}$ was the reduction for upper catchment sub‐stocks (CT), and a weak negative effect of Lat and weak positive effect of CPA were also present. For $h^{*}$, all covariates had a positive coefficients with strong effects of CPA and LU in particular. When translated to the response scale intercepts terms, representative of whole catchment stocks with mean covariate values, were 3.76 (90% interval 1.97–6.85) for $S^{*}$and 0.24 (90% interval 0.17 to 0.33) for $h^{*}$.

### 3.2 Predictions of the best fit model

**FIGURE 4:**
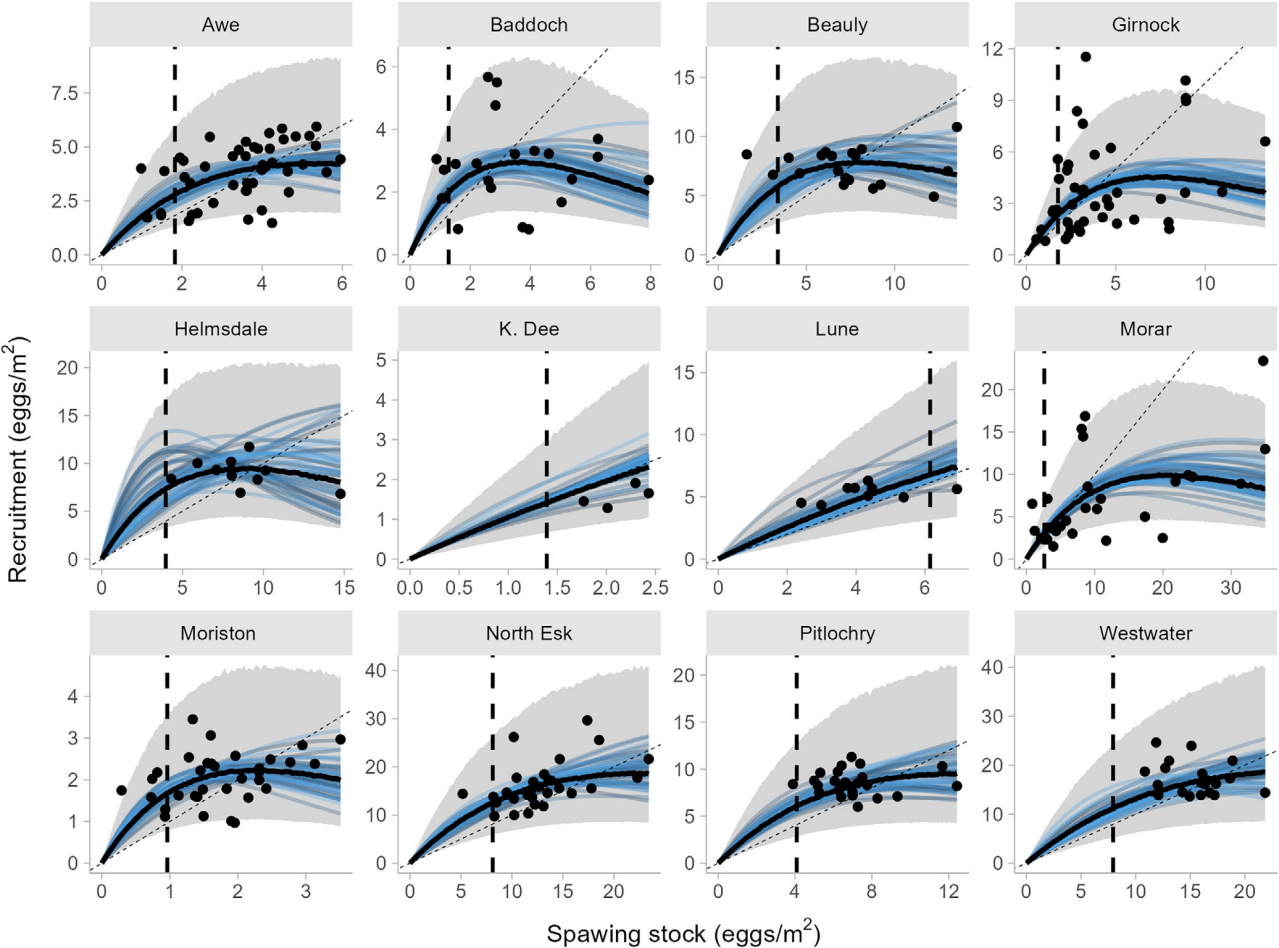
Fitted stock–recruitment (SR) relationships for 11 Scottish salmon stocks and 1 English stock (Lune), together with corresponding SR data (black points) resulting from $m^{*}$. The black solid lines and shaded area represent the posterior predictive median and 90% predictive intervals for new observations, conditional on the stock‐level effects and residual variance. The blue lines represent posterior simulations of the (latent) mean response. Dashed vertical lines indicate the median $S^{*},$ and the dashed diagonal lines indicate where recruitment is equal to spawning stock.

The fitted SR relationships together with the SR data are presented in Figure 4. For stocks with weakly informative data, such as the Lune and the K. Dee, the hierarchical structure results in shrinkage of $S^{*}$ and $h^{*}$ towards the common mean. This was particularly evident in the non‐zero median $S^{*}$ for the K. Dee, which an independent Ricker model would struggle to estimate given that all four data points lie below the line where recruitment is equal to spawning stock size (Figure 4). Thus, the hyper‐parameter estimates and choice of explanatory variables were not only important for out‐of‐sample stocks but also informative for within‐sample estimates.

**FIGURE 5:**
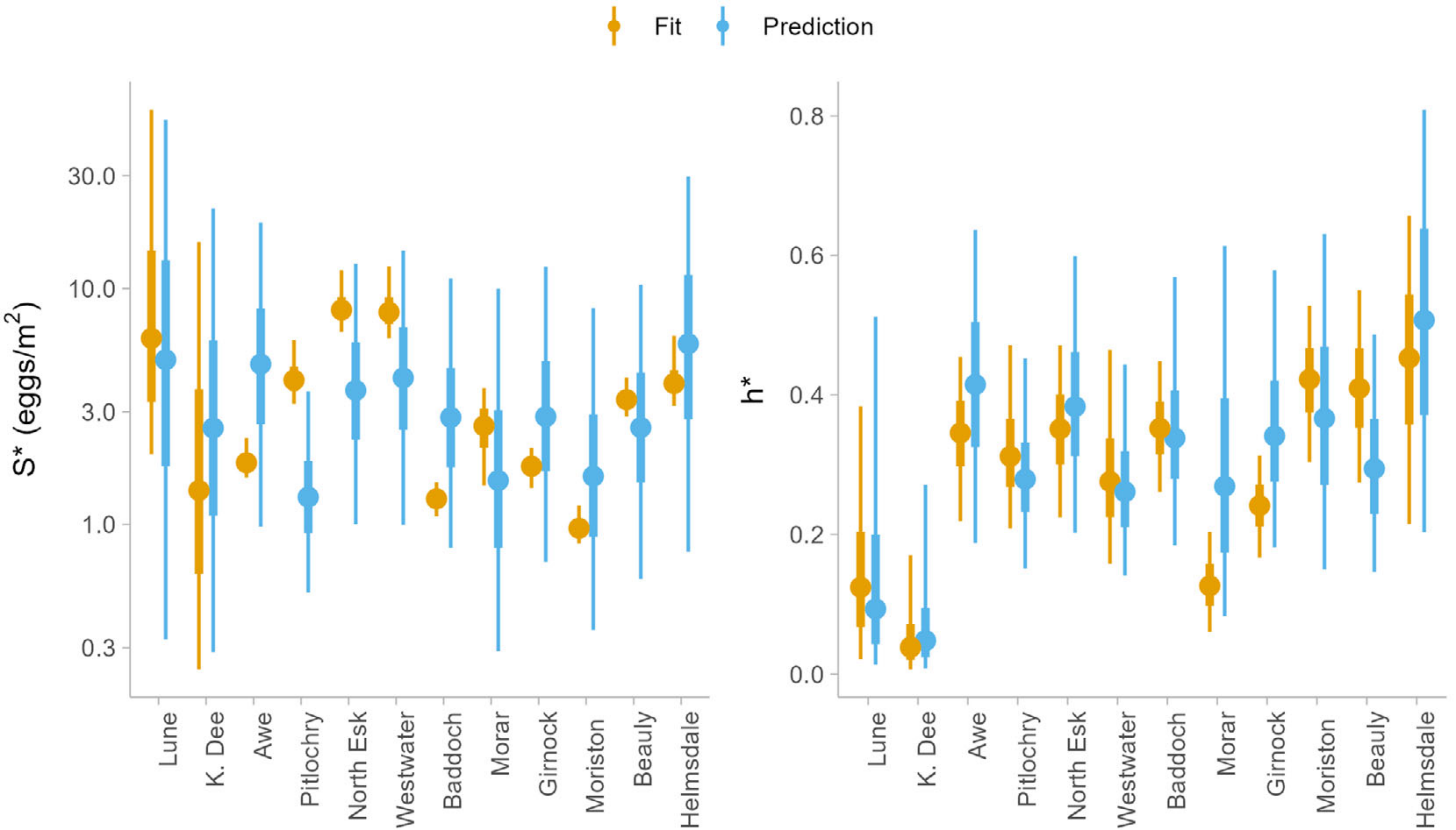
Median, 50% and 90% intervals of spawning stock ($S^{*}$) and harvest rate ($h^{*}$) at maximum sustainable yield (MSY) for each monitored stock ordered by latitude. Orange indicates fit values from final model using all data, and blue indicates predictions for left‐out stocks from the leave‐one‐stock‐out cross validation.

Figure 5 shows the fitted $S^{*}$ and $h^{*}$ for each stock from $m^{*}$ fit on all data together with predictions from $m^{*}$ for that stock when that stock left out of the data during the leave‐one‐stock‐out process. Generally, the predictions for left‐out stocks encompass the fitted values, indicative of good out‐of‐sample predictive performance, though the median value $S^{*}$ for Pitlochry (River Tummel upper catchment) falls above the 90% posterior prediction credible interval. Uncertainty in predicted $S^{*}$ was large relative to the fitted values, with the exception of the Lune and K. Dee stocks where the small number of available data points results in large uncertainty in the fitted values also. These weakly informative stocks fall at the lower extreme of the range of Lat and CPA and upper extreme of LU for the monitored stocks (Table 1), consequently predictions of $S^{*}$ for new stocks at this area of the covariate space will have large uncertainty. The fit $h^{*}$ values translate to low recruitment rates per spawner at MSY, $\frac{R^{*}}{S^{*}}=\frac{1}{\left(1-h^{*}\right)}$, with median (5%–95% CI) estimates ranging from 1.04 (1.02–1.08) for the K. Dee to 1.83 (1.56–2.19) for the Helmsdale.

**FIGURE 6:**
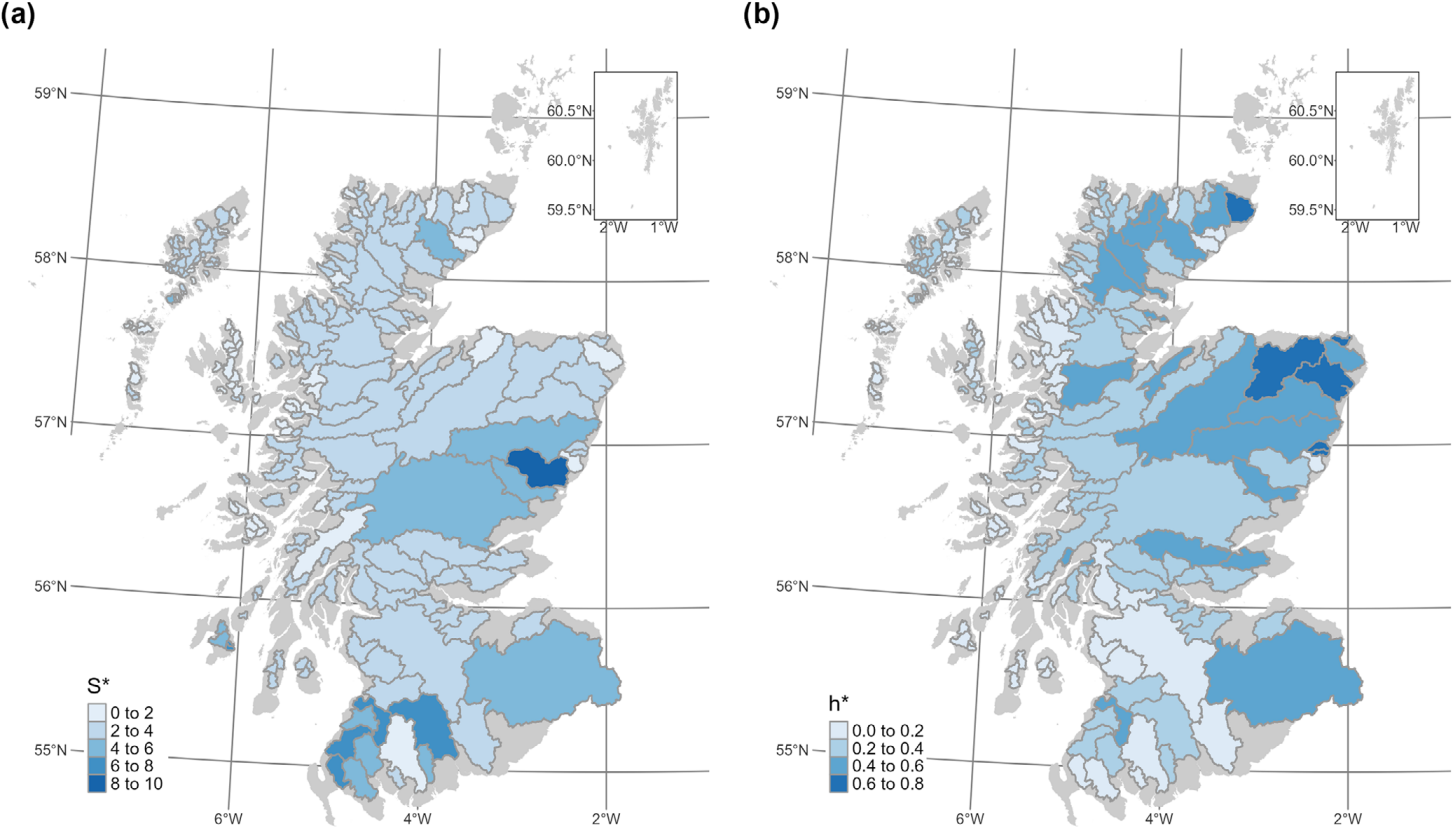
Median predicted $S^{*}$ (a) and $h^{*}$ (b) for all assessment areas. Salmon Stock Assessment Area boundaries derived from the Scottish Environment Protection Agency (SEPA) 1:50,000 river catchment data. Based on digital spatial data licensed from the UK Centre for Ecology and Hydrology, © NERC (CEH). Contains Ordnance Survey data © Crown copyright and database right 2024.

When transporting to out‐of‐sample stocks, the median posterior predicted $S^{*}$ ranged from 0.83 to 7.2 eggs/m^2^ (Figure 6), with 5% percentiles between 0.5 and 1.3 eggs/m^2^ and 95% percentiles between 6.7 and 53 eggs/m^2^.

## 4 DISCUSSION

The BHSR method presented provides a consistent framework for the joint analysis of salmon stocks, pooling valuable stock‐recruitment information across monitored populations. It allows for the specification of a relationship across key hyper‐parameters of recruitment models which facilitates the transportation of BRPs to non‐monitored populations for the purposes of fisheries management. The present analysis used previous BHSR frameworks (Prévost et al., 2003; White et al., 2016) with additional explanatory covariates complemented by a robust methodology for model selection that aims to maximise predictive performance when transporting BRPs. This approach can readily be applied within other fisheries management situations.

Among‐stock variation in $S^{*}$ has been found to be significant in prior analyses of monitored salmon stocks (Chaput et al., 1998; Prévost et al., 2003; White et al., 2016) and was also evident here (Figure 5). This leads to uncertainty in posterior predictions and motivates the inclusion of variables that may explain some of the variance. A significant limitation on the selection of explanatory variables is the requirement that they be available and consistent for all stocks including those for which predictions are required. Identifying such variables is likely to be more feasible by undertaking the analysis at a national, rather than international, scale. The national and multivariate focus of this study enabled the selection of a higher dimensional model than previous single covariate approaches (Prévost et al., 2003) and has the flexibility to predict different values of $S^{*}$ for stocks with similar latitudes but that differ in other metrics (Figure 6). The model selection process identified a model for Scottish stocks which included covariates of latitude, CPA, land use and catchment type.

Latitude has previously been shown to be an effective proxy for recruitment at relatively large scales, showing a positive relationship (Prévost et al., 2003; White et al., 2016). Here, a positive relationship between latitude and $h^{*}$, and by extension recruitment per spawner, $\frac{R^{*}}{S^{*}}=\frac{1}{\left(1-h^{*}\right)}$, was reproduced but a weak negative relationship between latitude and $S^{*}$ was found (Figure 3). This may be explained by the presence of other covariates in a multivariate context complicating the interpretation of the coefficient on latitude. In particular, CPA is assumed to capture relative productivity between the monitored stocks and is correlated with latitude (⍴ = 0.68). CPA has a positive relationship with both $S^{*}$ and $h^{*}$ and may be a better proxy for productivity rather than latitude at the national scale.

The productivity of a river is dependent on the amount and quality of freshwater habitat available for juvenile rearing which is influenced by both landscape and anthropogenic land use (Ugedal & Finstad, 2011). The densities of juvenile salmon in Scotland, and elsewhere, have been shown to be related to latitude and landscape variables (Malcolm et al., 2019). The use of a single metric to describe the land use of a catchment may not be sufficient to capture the complexities of the processes linking habitat to productivity which operate within a series of hierarchically arranged spatial scales (Ugedal & Finstad, 2011). Quantification of salmon habitat quality with respect to recruitment at a sub‐catchment scale could provide such linkage, but such data are difficult and costly to produce in a standardised manner across hundreds of catchments requiring assessment. Nonetheless, in the present analysis the broad distinction between predominately mountainous or heathland catchments in the north and northeast of Scotland and predominately grassland catchments in the south and southwest captured by the land usage metric had high utility when predicting the productivity of unobserved stocks.

Upper catchment stocks were found to have to lower $S^{*}$ compared to whole catchments (Figure 3). In Scotland, the habitat used by these stocks tends to have different physical and biological characteristics to that used by stocks inhabiting lower catchment areas. In addition to altitudinal differences, there are known to be differences in the biological characteristics of adult and juvenile salmon (Bacon et al., 2012; Stewart et al., 2006) as well as productivity between upper and lower catchments (Malcolm et al., 2019). It is therefore unsurprising that within‐catchment differences in $S^{*}$ exist.

When considering a range of explanatory variables, there is a risk of overfitting to the data and poor out‐of‐sample prediction (Wenger & Olden, 2012). This motivates the use of a blocked CV method that explicitly considers the likelihood of data for stocks that were not used in the model fitting process, thus aligning with the application for management (Hanson et al., 2020). When testing a non‐linear approach to modelling spatial variation this was rejected in favour of the simpler linear latitudinal relationship (Supplementary Material in Data S1, Figure 2). In general, more complex models tend to have poorer generality or transferability due to overfitting on the training data, which likely explains the rejection of the smoother (Wenger & Olden, 2012). The philosophy behind the CV model selection method used here emphasises out‐of‐sample prediction over providing inference into ecological processes. Such an approach is considered appropriate for management applications and can be beneficial for prediction within complex systems (DeAngelis & Yurek, 2015; Ye et al. 2015). However, using the same data to select a model as for fitting can result in biased estimates or optimistic confidence intervals (Yates et al., 2023). Caution should be taken, therefore, against over interpretation of the relative effect sizes of the covariates while recognising their predictive utility (Figure 2).

The choice of which part of the life cycle is used as the recruitment axis of the SR relationship will have an impact on the prediction of $S^{*}$. Where MSY is being used as a target to maximise fisheries yield then recruits should be prior to fisheries (pre‐fishery abundance), though in the case of setting conservation limits for salmon, MSY is not being used as a target in the traditional manner but rather as a limit that should not be dropped below (Potter et al., 2003). Using recruit data after the fisheries take will lead to a lower SR curve giving a lower S* compared to pre‐fishery recruit abundance, with the extent of the difference being related to the level of fishery take. Production of pre‐fishery abundance estimates for upper tributary sites is complex as many do not contain fisheries even though an unknown number of fish destined for these sites will be exploited downstream of the trap/counter. Instead, it was decided to undertake the analysis using spawner to spawner data (converted to eggs), giving access to a larger number of sites with the aim of more fully representing the variety seen within Scotland. The (median) posterior predictions of $S^{*}$ for out‐of‐sample stocks ranged between 0.83 and 7.2 (Figure 6) and, despite these caveats, were similar to those reported in neighbouring jurisdictions: Republic of Ireland, median $S^{*}$ for out‐of‐sample stocks approx. 2–5 eggs/m 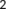 (White et al., 2016); England and Wales, egg targets 0.7–4.0 eggs/m 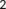 [Centre for Environment, Fisheries & Aquaculture Science (CEFAS 2021)]. The analysis of Prévost et al. (2003) estimated larger median $S^{*}$ values of 4.9 to 12.9 eggs/m 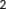 for out‐of‐sample rivers across the latitudinal range of Scotland (55 to 60 degrees). However, direct comparison with our approach is difficult given differences in the underlying data used by Prévost et al. (2003) which considers only two Scottish rivers, limits data to post 1980 and calculates recruitment from returns to home waters (as opposed to spawners) under various assumptions regarding marine mortality (Crozier et al., 2003). The additional mortality included in recruitment estimated at spawning may partially account for the reduced $S^{*}$ values in the present analysis.

There remain multiple avenues to improve the methods to derive conservation limits. Of particular interest is the exploration of different functional relationships used to model recruitment, which can impact the MSY point and therefore conservation limits (Barrowman & Myers, 2000; Forrest et al., 2010; Michielsens & McAllister, 2004; Walters & Korman, 2001). The parameterisation used here implicitly assumes a positive harvest rate, which forces recruitment to exceed replacement for low spawning stock. However, endangered stocks may not be able to achieve replacement, making such assumptions invalid. Despite being within the predictive ability of the model presented here, all data for the K. Dee fall below the replacement line and may be better captured by models that allow for recruitment below replacement. Fundamentally, conservation requires an understanding of the current and future status of stocks. Anadromous fish populations can exhibit substantial interannual fluctuations in abundance, which may be indicative of shifts in productivity (Chaput et al., 2005; Hilborn, 2001) and non‐linear dynamics (DeAngelis & Yurek, 2015). Consideration of the residuals of the final model indicates that Scottish stocks may have been subject to such productivity shifts (Supplementary Material in Data S1). These aspects of population dynamics are notoriously hard to forecast (Holt & Michielsens, 2020; Subbey et al., 2014) and not addressed by the present analysis. The use of data dating back to 1963 for some stocks raises the potential for estimates of $S^{*}$ to be biased by recruitment under different productivity regimes and not reflective of current conditions. Future efforts should be considerate of long‐term productivity shifts and where appropriate restrict the temporal range of SR data (Prévost et al., 2003) or explore time‐varying productivity when modelling (Holt & Michielsens, 2020).

However, perhaps the most important goal for management should be to improve the quantity and quality of stock–recruitment data. Extending the time‐series of weakly informative stocks such as the K. Dee and Lune will improve BRP estimates. The inclusion of data from a wider group of stocks will provide further insight into how BRPs vary among stocks and potentially enable consideration of more complex model structures, for example, non‐constant variance (Supplementary Material in Data S1). The extent to which the data used to make predictions are representative is a key issue for predicting out‐of‐sample. In an ideal scenario, monitoring would encompass the full range of environments of all managed stocks, but in reality this is rarely feasible due to resource limitations and the practical difficulties in obtaining stock recruitment data. In the present analysis, the range of the covariates in the selected model (Lat, LU and CPA) for the monitored stocks was reasonably representative, covering 82%, 74% and 100% of the out‐of‐sample stocks respectively. The range in wetted area of the monitored stocks covered 82% of the out‐of‐sample stocks; however, given the skewed distribution in catchment size, four of these stocks represent outliers in wetted area, having 2–4.5 times the area of the largest monitored stock. It is possible that the fitted relationships break down for such systems; issues with representativeness are a common consideration for studies which aim to extrapolate to out‐of‐sample stocks (Prévost et al., 2003; White et al., 2016).

## AUTHOR CONTRIBUTIONS

James P. Ounsley and Stuart J. Middlemas performed the formal analysis. Stuart J. Middlemas, Gordon W. Smith, Jonathan P. Gillson and Brian A. Shields curated the data. James P. Ounsley, Nora N. Hanson and Stuart J. Middlemas wrote the original draft. All authors contributed to reviewing and editing the manuscript.

## Supporting information


**Data S1.** Supporting information.

## Data Availability

Data available on request. All codes to run the models and cross validation are available at the Scottish Government Marine Directorate Github (https://github.com/MarineScotlandScience/salmon-cl).
