# Supplementary material for: Bayesian hierarchical stock–recruitment models for setting conservation limits for Atlantic salmon stocks in Scotland
Source: J Fish Biol. 2026 Feb 18;108(6):1988–99. doi: 10.1111/jfb.70363 (PMC13357261; doi:10.1111/jfb.70363)
Supplement: Supplementary file 1 — Data S1. Supporting information. [file JFB-108-1988-s001.docx]

Supplementary material

# Principal component analysis on land usage

A principal components analysis was used to reduce the 10 different variables from the 2015 Land Cover Map produced by the UK Centre for Ecology and Hydrology (Rowland et al., 2017) into a single covariate covering each stock of concern for fisheries management in Scotland. The first principal component, which explained 60% of the variation in land-use. Retained loadings of the first principal component broadly contrast assessment areas between mountainous and grassland regions (Table S1).

Table S1. variables and loadings of first principal component of land usage (LU) data mapped to assessment areas.

| Variable | PC1 Retained | PC1 Loading |
| --- | --- | --- |
| Mountain, heath and bog | Yes | -0.90 |
| Semi-natural grassland | Yes | 0.28 |
| Improved grassland | Yes | 0.22 |
| Coniferous woodland | Yes | 0.21 |
| Arable and horticultural | Yes | 0.12 |
| Broadleaf woodland | No | NA |
| Saltwater | No | NA |
| Freshwater | No | NA |
| Coastal | No | NA |
| Built-up areas and gardens | No | NA |

# Non-linear spatial variation in BRPs

Latitude has previously been shown to be an effective proxy for recruitment at relatively large scales (Prévost et al. 2003; White et al. 2016). However, more complex non-linear approaches to modelling spatial variation have also been found to improve within-sample model fit when explaining SR dynamics in other salmonids (Su et al. 2004). Given the complex geography of Scottish catchments, and the difference among those at the same latitude which drain from different sides of the country (Soulsby et al. 2009), we tested a more flexible approach to capturing spatial heterogeneity than a simple linear latitudinal relationship.

The proximity of the rivers or tributaries supporting each stock was represented via a spatial position metric (SP), constructed to capture broad topographic features of the Scottish coastline. Conceptually, this corresponds to a piecewise linear transformation of latitude of the mouth of the river supporting each stock (or counter / trap for monitored stocks): increasing with latitude along the east coast up to the northernmost river, and then continuing southward along the west coast by adding the absolute latitudinal difference from the northernmost point. Specifically, for rivers on the east coast, the metric is simply the latitude of the river mouth, counter or trap minus 54.5^∘ (minimum latitude in the SR dataset). After reaching the north coast of mainland Scotland, the metric becomes the east coast latitudinal distance (58.62–54.50) added to the absolute difference between the latitude of the west coast river mouth and 58.62 (Figure 1d). This results in a one-dimensional measure of location that captures information about the proximity of stocks around Scotland, embedding east and west coast stocks at different ends of a continuum.

The spatial position metric was used as the basis of a Bayesian penalised regression spline (P-spline, Lang and Brezger 2004). This was integrated into the BHSR model using a general additive model structure (Wood 2017). For models including SP, equation 1 from the main text was updated to the following

$$\text{log}(S_{g}^{*})\sim\text{Normal}(\boldsymbol{\beta}^{S}\mathbf{X}_{g}+\boldsymbol{\gamma}^{S}\mathbf{Z}_{g},\sigma^{S}),$$

Where $\mathbf{Z}_{g}$ is the design matrix for a set of B-spline basis functions of order 3 with j = 1,…,K knots, with corresponding coefficients $\boldsymbol{\gamma}^{S}$ (Wood 2017). To avoid overfitting, $\boldsymbol{\gamma}^{S}$ were penalised against excessive flexibility through the use of the 2^nd^ order difference random walk prior,

$$\gamma_{j}^{S}\sim\text{Normal}\left( 2\gamma_{j-1}^{S}-\gamma_{j-2}^{S},\tau^{S} \right)$$

with the hyperparameter $\tau^{S}$ controlling the freedom of the P-spline.

Equation 2 was updated in a similar way,

$$\text{logit}(h_{g}^{*})\sim\text{Normal}(\boldsymbol{\beta}^{h}\mathbf{X}_{g}+\boldsymbol{\gamma}^{h}\mathbf{Z}_{g},\sigma^{h}),$$

where $\boldsymbol{\gamma}^{h}$ are the coefficients for the basis functions on $h^{*}$. Note that $\mathbf{Z}_{g}$ is shared in the definition of $S^{*}$ and $h^{*}$. An identical 2^nd^ order difference random walk prior was also applied to $\boldsymbol{\gamma}^{h}$ with the hyper-parameter $\tau^{h}$ controlling the freedom of the P-spline.

Weakly informative standard normal priors where used for both $\boldsymbol{\gamma}^{S}$ and $\boldsymbol{\gamma}^{h}$ and half-Cauchy priors were used for $\tau^{S}$ and $\tau^{h}$ (Table S2).

For both P-splines, nine B-spline basis functions were used over a set of K=13 evenly spaced knots to allow sufficient flexibility to capture the spatial structure of the data. To transport BRPs to out-of-sample stocks, it was necessary to extend the lower range of the knots from the minimum SP of stocks with SR data (2.19) to the minimum of all stocks (1.26). Consequently, estimates of the spline below 2.19 are extrapolations.

Table S2 Priors on parameters and hyper parameters in the model including the Bayesian P-spline.

| (Hyper) parameter | Bounds | Prior |
| --- | --- | --- |
| $\boldsymbol{\beta}^{S}$ | +/- inf. | Normal(0,1) |
| $\boldsymbol{\beta}^{h}$ | +/- inf. | Normal(0,1) |
| $\boldsymbol{\gamma}^{S}$ | +/- inf. | Normal(0,1) |
| $\boldsymbol{\gamma}^{h}$ | +/- inf. | Normal(0,1) |
| $\tau^{S}$ | 0, +inf. | Half-Normal(0,1) |
| $\tau^{h}$ | 0, +inf. | Half-Normal(0,1) |
| $\sigma^{S}$ | 0, +inf. | Half-Cauchy(0,1) |
| $\sigma^{h}$ | 0, +inf. | Half-Cauchy(0,1) |
| $\sigma$ | 0, +inf. | Half-Cauchy(0,1) |

# Model diagnostics

## Residuals

Median residuals, derived on the log scale, against median fitted values on the log scale from the final model are shown in Figure S1. The residuals show no obvious systematic bias, though in Figure 4 in the main text there is evidence of non-constant variance between stocks, with the Girnock, Baddoch and Morar having noticeably greater error in the residuals. In preliminary analysis not presented a hierarchical / partial pooling implementation of the variance parameter was attempted to account for this eventuality, however this resulted in significant convergence issues and was not taken forward. This was likely due to an insufficient number of groups to fully identify the variance.

As a test of the sensitivity of the model to the complete pooling of variance assumption we refit the final model but with the extreme assumption of completely independent variance between stocks. Whilst this allows testing of the complete pooling variance structure, this approach would not enable generation of variance for new stocks and therefor preclude likelihood estimates for new data and the possibility of cross validation. Posterior estimates of the variance for each stock together with the variance under the complete pooling assumption are shown in Figure S2. This independent variance model reinforces that the Girnock, Baddoch and Morar have greater variance than the other stocks, which largely overlap in their variance estimates. Given the divergence of the independent variance estimates, there is no substantial overlap between the stock level variance estimates and the complete pooling variance estimate.

Effect sizes for covariates of this model compared with the complete pooling model are shown in Figure S3. Effect sizes are reasonably consistent, however there is some general shrinkage and a noticeably diminished latitudinal effect in the independent variance model. This indicates that there may be some value in structuring the variance but this is ultimately limited due to data availability and the requirement to predict for new stocks. Although residual variances differ among stock, our predictive focus is on the latent mean effects for new stocks. Observation-level variance enters the model only as a nuisance parameter and is integrated out when forming predictions of BRPs. Excepting the above impacts on effect size estimates, heteroskedasticity primarily affects predictive uncertainty for observations.

Figure S4 shows the residuals on the log scale by year. There is some evidence of a temporal pattern in the residuals across all stocks, which may warrant consideration of stock recruitment models that can account for temporal variation in future analysis, as noted in the discussion.


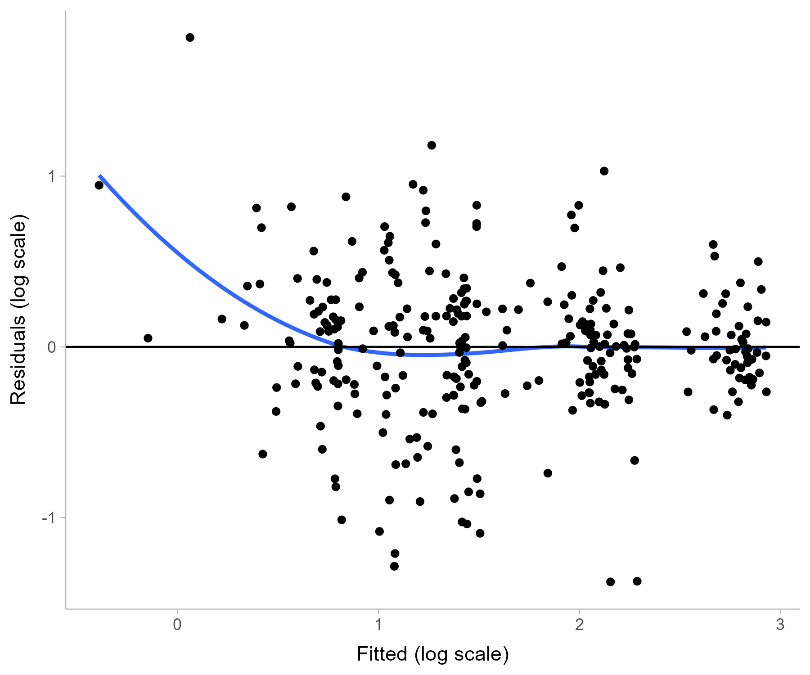


Figure S1 Median residuals against fitted values on the log scale from m*, with a LOESS smooth (blue line).


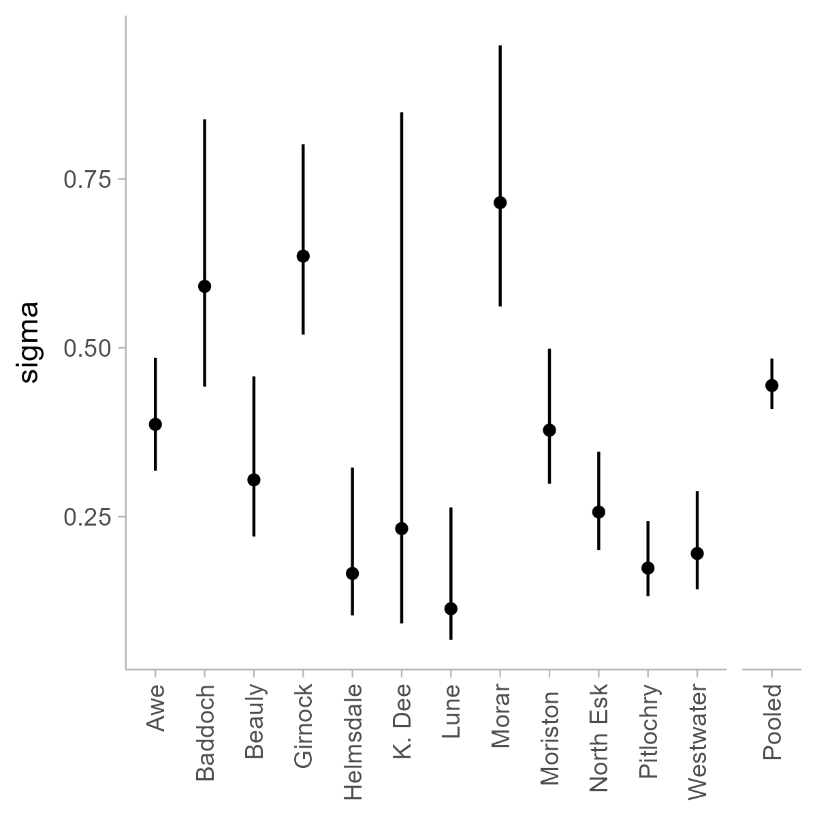


Figure S2 Median and 95% percentiles of the variance parameter for each stock from an independent variance model and the complete pooling model presented in the main text.


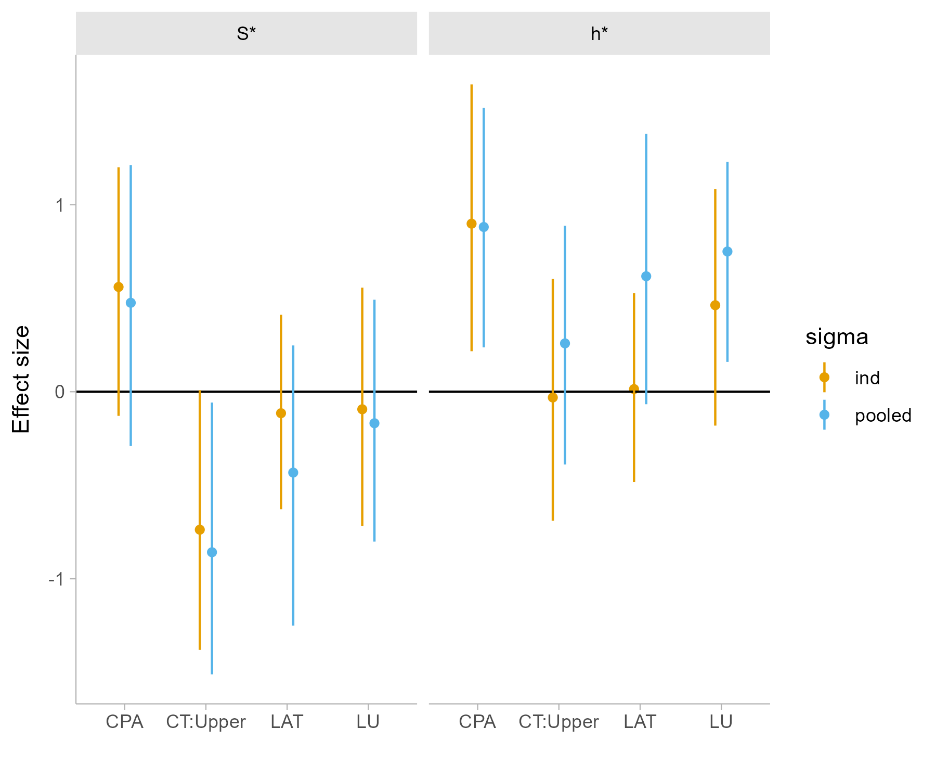


Figure S3 Posterior median and 90% intervals for effect on each covariate on the linear predictor scale. Estimates under the complete pooling of variance assumption (blue) and independent variance assumption (orange) are compared.


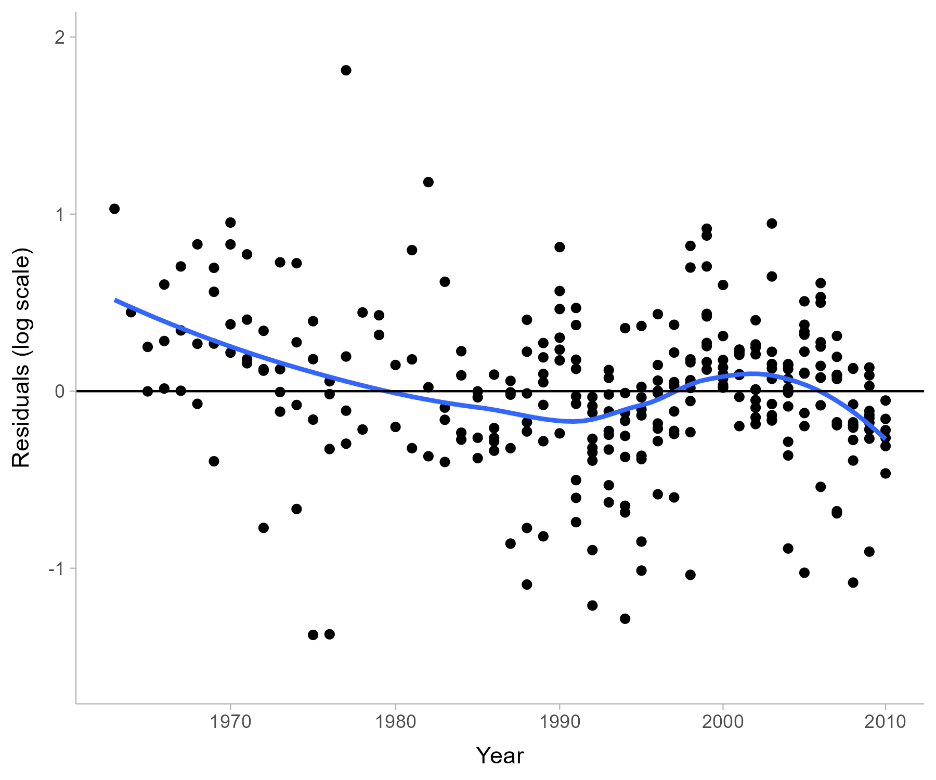


Figure S4 Median residuals on the log scale by year with a LOESS smooth (blue line).

## Parameters

Median and 95% summary statistics for the prior and posterior of each unknown in the model are presented in Table S2 and a correlation matrix between the posterior samples of the coefficients on the linear predictors are shown in Table S3. These summaries indicate that the data are informative for the parameters and there is no strong confusion between covariates (with the largest correlation being .52).

| Parameter | Covariate | Distribution | Median | 2.5% | 97.5% |
| --- | --- | --- | --- | --- | --- |
| $\boldsymbol{\beta}^{\boldsymbol{S}}$ | Intercept | prior | 0 | -1.96 | 1.96 |
| $\boldsymbol{\beta}^{\boldsymbol{S}}$ | Intercept | post | 1.32 | 0.68 | 1.92 |
| $\boldsymbol{\beta}^{\boldsymbol{S}}$ | CPA | prior | 0 | -1.95 | 1.95 |
| $\boldsymbol{\beta}^{\boldsymbol{S}}$ | CPA | post | 0.48 | -0.45 | 1.36 |
| $\boldsymbol{\beta}^{\boldsymbol{S}}$ | LU | prior | 0 | -1.97 | 1.97 |
| $\boldsymbol{\beta}^{\boldsymbol{S}}$ | LU | post | -0.17 | -0.94 | 0.65 |
| $\boldsymbol{\beta}^{\boldsymbol{S}}$ | CT | prior | -0.01 | -1.95 | 1.96 |
| $\boldsymbol{\beta}^{\boldsymbol{S}}$ | CT | post | -0.86 | -1.64 | 0.12 |
| $\boldsymbol{\beta}^{\boldsymbol{S}}$ | LAT | prior | 0 | -1.97 | 1.97 |
| $\boldsymbol{\beta}^{\boldsymbol{S}}$ | LAT | post | -0.43 | -1.43 | 0.38 |
| $\boldsymbol{\beta}^{\boldsymbol{h}}$ | Intercept | prior | 0 | -1.97 | 1.97 |
| $\boldsymbol{\beta}^{\boldsymbol{h}}$ | Intercept | post | -1.15 | -1.73 | -0.6 |
| $\boldsymbol{\beta}^{\boldsymbol{h}}$ | CPA | prior | 0 | -1.95 | 1.94 |
| $\boldsymbol{\beta}^{\boldsymbol{h}}$ | CPA | post | 0.88 | 0.09 | 1.65 |
| $\boldsymbol{\beta}^{\boldsymbol{h}}$ | LU | prior | 0 | -1.97 | 1.97 |
| $\boldsymbol{\beta}^{\boldsymbol{h}}$ | LU | post | 0.75 | 0.01 | 1.33 |
| $\boldsymbol{\beta}^{\boldsymbol{h}}$ | CT | prior | 0 | -1.97 | 1.97 |
| $\boldsymbol{\beta}^{\boldsymbol{h}}$ | CT | post | 0.26 | -0.53 | 1.01 |
| $\boldsymbol{\beta}^{\boldsymbol{h}}$ | LAT | prior | 0 | -1.98 | 1.96 |
| $\boldsymbol{\beta}^{\boldsymbol{h}}$ | LAT | post | 0.62 | -0.21 | 1.53 |
| $\boldsymbol{\sigma}$ | - | prior | 1 | 0.04 | 26.46 |
| $\boldsymbol{\sigma}$ | - | post | 0.44 | 0.41 | 0.48 |
| $\boldsymbol{\sigma}_{\boldsymbol{S}}$ | - | prior | 1 | 0.04 | 23.79 |
| $\boldsymbol{\sigma}_{\boldsymbol{S}}$ | - | post | 0.62 | 0.36 | 1.19 |
| $\boldsymbol{\sigma}_{\boldsymbol{h}}$ | - | prior | 1 | 0.04 | 26.5 |
| $\boldsymbol{\sigma}_{\boldsymbol{h}}$ | - | post | 0.23 | 0.01 | 0.82 |

Table S2 Summary of prior and posterior distributions sampled via MCMC for parameters of the BHSR model.

|  | | h* | | | | S* | | | |
| --- | --- | --- | --- | --- | --- | --- | --- | --- | --- |
|  |  | **CPA** | **LU** | **CT** | **LAT** | **CPA** | **LU** | **CT** | **LAT** |
| h* | **CPA** | 1 | 0.5 | -0.14 | -0.1 | 0.06 | 0.03 | 0.08 | -0.08 |
|  | **LU** | 0.5 | 1 | 0.33 | 0.36 | 0.01 | -0.03 | -0.04 | -0.05 |
|  | **CT** | -0.14 | 0.33 | 1 | 0.09 | 0.06 | -0.01 | -0.06 | 0.01 |
|  | **LAT** | -0.1 | 0.36 | 0.09 | 1 | -0.11 | -0.07 | -0.04 | -0.19 |
| S* | **CPA** | 0.06 | 0.01 | 0.06 | -0.11 | 1 | 0.52 | -0.02 | -0.09 |
|  | **LU** | 0.03 | -0.03 | -0.01 | -0.07 | 0.52 | 1 | 0.31 | 0.4 |
|  | **CT** | 0.08 | -0.04 | -0.06 | -0.04 | -0.02 | 0.31 | 1 | 0.21 |
|  | **LAT** | -0.08 | -0.05 | 0.01 | -0.19 | -0.09 | 0.4 | 0.21 | 1 |

Table S3 Posterior correlation matrix for coefficients on the effect of each covariate on S* and h*.

**References**

Lang, S., and Brezger. A. 2004. Bayesian P-Splines. Journal of Computational and Graphical Statistics, 13(1): 183–212.

Prévost, E., Parent, E., Crozier, W., Davidson, I., Dumas, J., Gudbergsson, G., Hindar, K., McGinnity, P., MacLean, J., and Sættem, L. M. 2003. Setting biological reference points for Atlantic salmon stocks: Transfer of information from data-rich to sparse-data situations by Bayesian hierarchical modelling. ICES Journal of Marine Science 60 (6): 1177–1193.

[dataset]* Rowland, C.S., Morton, R.D., Carrasco, L., McShane, G., O’Neil, A.W., Wood, C.M. 2017. Land Cover Map 2015 (1km percentage target class, GB). NERC Environmental Information Data Centre.

Soulsby, C., Malcolm, I. A., Tetzlaff, D. and Gibbins, C.N. 2009. Chapter 10 - British and Irish Rivers. In Rivers of Europe. Ed. by Tockner, K., Uehlinger, U. and Robinson, C. T., 381–419. Academic Press, London. Su, Z., Peterman, R. M., and Haeseker, S. L. 2004. Spatial hierarchical Bayesian models for stock-recruitment analysis of pink salmon (Oncorhynchus gorbuscha). Canadian Journal of Fisheries and Aquatic Sciences, 61(12), 2471–2486.

White, J., Ó Maoiléidigh, N., Gargan, P., de Eyto, E., Chaput, G., Roche, W., McGinnity, P., Crozier, W. W., Boylan, P., Doherty, D., O'Higgins, K., Kennedy, B., Lawler, I., Lyons, D., Marnell, F. 2016. Incorporating natural variability in biological reference points and population dynamics into management of Atlantic salmon (Salmo salar L.) stocks returning to home waters. ICES Journal of Marine Science 73 (6): 1513–24.

Wood, S. N. 2017. Generalized additive models: an introduction with R. Chapman and Hall/CRC Press, London.
